# Authentic content as an effective mechanism for embedding patient voices in health innovation: a hypothesis and conceptual framework

**DOI:** 10.3389/frhs.2026.1833920

**Published:** 2026-09-18

**Authors:** Rohin Malhotra

**Affiliations:** Boofel Enterprises, London, United Kingdom

**Keywords:** accelerating innovation, artificial intelligence, authentic content, clinical trials, health innovation system, patient voice, ROI, stakeholder engagement

## Abstract

Health innovation systems continue to face persistent inefficiencies, mistrust, and escalating development costs despite rapid scientific progress. Although patient-engagement is increasingly recognised as essential for patient-centred healthcare innovation, there is limited conceptual and empirical research explaining how patient voices can be systematically embedded within processes and it is relatively unexplored in literature how this influences measurable outcomes such as engagement, collaboration, trust and return-on-investment. This paper addresses this gap through a hypothesis and conceptual analysis informed by interdisciplinary literature, industry reports and case-based evidence examining the integration of patient voices across health innovation processes and posits that authentic content functions as an effective and scalable mechanism for embedding patient voices across multiple stages of the health innovation lifecycle. The emergence of artificial intelligence also offers the potential to increase and amplify diffusions from authentic content and facilitate mass personalisation of patient voices across innovation lifecycles but, even without this, authentic content is proposed as a strategic priority and a high-fidelity mechanism for multi-level stakeholder engagement that may be associated with enhanced patient access and stronger financial outcomes. The timing of authentic content deployment is also hypothesised to be an important determinant of its potential impact, with earlier integration proposed to produce a “leftward shift” in the cumulative trajectory of stakeholder-engagement and value-creation and an acceleration of development that, if empirically supported, may be associated with longer periods of effective commercial market exclusivity, faster patient access to medicines, and more efficient provision of access through greater scalability. If validated through future empirical research and academic rigour, authentic content may represent a scalable and measurable approach for embedding patient voices as a strategic driver of engagement, innovation efficiency, and patient-centred value creation in health innovation systems.

## 1 Introduction

### 1.1 A problem of costly health innovation in need of measurable solutions

Global health innovations are advancing with unprecedented pace, yet translation of scientific discoveries into patient value is hindered by inefficiencies, mistrust and unsustainable costs. Bringing new pharmaceutical assets to market is estimated to cost $2.3 billion (1) and the “last mile” of value capture is fraught with inefficiencies (2). 85% of clinical trials fail to retain sufficient patients (3), public trust in pharma is low, and non-adherence drains over $528 billion annually in the US alone (4). Significant portions of this “cost of failure” are preventable by addressing misalignments between clinical design, patient realities or adoption of better patient-centric strategies (5).

There are efforts to incorporate the patient's voice and experience into health innovation processes and for more patient-focused drug development (6), with industry reports highlighting patient-centricity as being crucial for successful innovation (7). However, tangible evidence of whether, and how, integration of patient voices drives quantifiable outcomes is needed to elevate such initiatives from anecdotal support to standard practice.

### 1.2 Embedding patient voices into health innovation

Integrating the patient voice into health innovation lifecycles marks a transition from viewing patients as passive subjects to recognising them as essential co-designers of medical progress (8). By addressing the lived experience of patients, it is possible that innovators can de-risk programs and streamline paths to market (9) and inclusion of patient voices offers a strategic opportunity for substantial cost-savings, a higher probability of commercial and clinical success, and increased likelihood for long-term treatment adherence (10).

Integration of patient voices into health innovation is not a singular event but a diverse spectrum of physical and methodological interventions in multiple ways. This occurs through the physical recruitment of patients into Patient Advisory Boards (where “patient experts” collaborate directly with clinical development teams), involvement in workshops, via academic research, in evidence-driven contexts like Patient-Reported Outcome Measures (PROMs), Social Media Listening (SML), physical digital touchpoints like Electronic Health Record (EHR) portals (11), and, increasingly, video content.

Video content is increasingly recognised as a high-fidelity source of qualitative evidence and phenomenological insight, with an ability to scalably capture non-verbal cues, emotional context and functional mobility that text-based reports cannot convey (12), but there are many types of, and ways to create and diffuse, video content and its ability to serve as an effective tool for embedding patient voices requires further consideration and analysis.

### 1.3 Authentic content provides an effective means for integrating patient voices

Academic literature recognises multiple categories of video content in healthcare. There are pedagogical and simulation-based videos (foundational to medical education, providing standardised, repeatable environments for clinical training without risking patient safety (13), immersive Virtual Reality content, strategic and promotional video content (often used to communicate complex scientific data and stakeholder understanding, and to promote solutions to patients with clinical and consumer-centric tonality), animated videos, curated videos that utilise actors to simulate or recreate experiences, and video content from authentic patients' and stakeholders’ lived experiences (“authentic content”).

Whilst all categories of video content can contribute to embedding patient voices in health innovation, this paper focuses on authentic content for three reasons. First, it is the only category in which the patient voice constitutes unmediated primary data rather than a third-party representation or reconstruction, making it the definitionally appropriate unit of analysis for a study of patient-voice integration (12). Second, evidence indicates that the effects of narrative interventions are conditional on story type and source (14), and that exposure to real patients' first-person experiences operates through distinct mechanisms (15, 16), warranting analysis of authentic content as a discrete category rather than subsuming it within video content generally. Third, whereas simulation-based, promotional and animated video are well-established within their respective literatures, authentic content remains under-examined as a systematic mechanism across the health innovation lifecycle—the gap this paper seeks to address.

Traditional market research treats patients as data, failing to capture emotional and logistical complexities of lived experiences, and processes can be binary, shaping plans and development around metrics and siloed care pathways (17), but in a world of caregivers, dependencies and comorbidities (18) authentic content can embed patients' [and other] voices and provide more dynamic understanding of patients' and stakeholders' experiences (19).

Embedding patient voices increases satisfaction, adherence and productivity, while helping organisations avoid costly inefficiencies (20)—providing evidence consistent with the proposition that patient-centred storytelling (12) may accelerate collaboration and innovation and act as a de-risking mechanism and value-multiplier. Real-life stories have emerged as effective tools that bring patient experiences to life for these stakeholders.

Experiences and perspectives from patient journeys, from symptoms to diagnosis to treatment and daily life for patients and caregivers, build shared understanding among manufacturers, healthcare professionals (HCPs), payers and other stakeholders. These authentic patient stories can highlight pain points and unmet needs more effectively than data alone, creating alignment on solving problems and a greater sense of collaboration towards shared goals that may enhance outcomes and ROI. Stakeholders identify more with real-life stories and connect with patient-centred rationale (21), which can galvanise support for innovative solutions or pathway changes, with potentially positive implications for ROI and outcomes (22).

### 1.4 Aim and structure

An aim of this paper is to advance a hypothesis and conceptual framework explaining how authentic content may function as a mechanism for embedding patient voices across the stages of health innovation systems, and to set out how that hypothesis can be operationalised and tested. The Hypothesis section defines authentic content, explains why authenticity is proposed to matter, describes the stakeholder-engagement mechanism through which authentic content is proposed to operate, introduces the Health Innovation System (HIS) Framework and situates the framework within established theoretical literatures. The subsequent section reviews evidence bearing on the hypothesis at each stage of innovation, prefaced by an explicit account of the tiers of evidence on which it draws. The Discussion then examines the timing of deployment, the emerging role of artificial intelligence, the operationalisation of the framework for empirical validation, and the limitations of the analysis, before the Conclusion summarises implications for research and practice.

## 2 The hypothesis

Authentic content provides an opportunity for structured and multi-level diffusion of patient voices and authentic patients' and stakeholders’ lived experiences across early and later stages of health innovation systems, and is hypothesised to increase engagement, collaboration, trust and return-on-investment (ROI), and to accelerate progression of development.

### 2.1 Why content needs to be authentic

To be an effective vehicle for patient voices, content must prioritise authenticity—defined here as the unscripted, lived experiences of real patients—over the polished, curated aesthetic often associated with traditional marketing that utilises actors, staged settings and storyboards.

The use of actors or highly curated scripts creates a “credibility gap” that undermines the fundamental purpose of healthcare communication. When patient narratives are perceived as manufactured, they trigger a scepticism that diminishes their utility in clinical and strategic planning. Authentic, raw video content serves as a form of narrative evidence that actors cannot replicate due to a lack of physiological and psychological nuances (23). Moreover, in specialised sectors such as Medical Affairs where the need for actionable insights relies on authenticity, curated content often sanitises the complexities of a disease to fit a brand narrative. Research suggests that when HCPs view unscripted patient testimonials, with editing that preserves the integrity of captured footage, there is a higher level of “empathic engagement” that directly influences prescribing behaviors and the adoption of medical innovations (24, 25) and a greater belief that it represents an accurate window into real-world adherence challenges and lifestyle impacts.

The “realness” of authentic content is a proxy for institutional transparency, reflecting the messy, non-linear reality of patient experiences and treatment, and so the use of actors or overly curated videos can be perceived as “patient-washing”—which leads to breakdowns in trust between innovators and communities they serve (14) and fails to meet the rigorous standards of qualitative evidence required by the modern medical landscape (26).

### 2.2 Authentic content provides a mechanism for collaboration

Although authentic content has been associated with the communication of real, dynamic, longitudinal lived experiences, deeper stakeholder-engagement, accelerated development, amplified learning, and improved financial and clinical outcomes (27), it is treated as a “soft” contributor rather than a core strategic input; arguably, its potential may be under-examined. Whilst increasingly recognised as a potentially effective driver of innovation (28), this still ignores a potentially effective role as a *stakeholder-engagement mechanism*.

In order to serve as this *mechanism*, content must feature real patients with no actors, scripts, recreations or curations (so it can still be aligned with regulatory, compliance, corporate or ethical rules without compromising authenticity). A key feature of authentic content from “patient journeys” is how it can, and should, also feature genuine stakeholders from those “journeys,” including loved ones, caregivers, physicians, nurses and HCPs. Indeed, it is possible to not feature patients at all (if designed to help HCPs face challenges with specific care pathways, content could legitimately feature only HCPs speaking about their patient-centric experiences).

The content-creation process is a dynamic stakeholder-engagement mechanism and recruitment of participants is critical. Once established that content will be authentic with real-life contributors and not “curated” (polished, scripted, storyboarded, staged and sanitized with brand-led tonality), this attracts stakeholders to become participants, recruitment partners, sponsors, supporters and distributors—and this leads to a mechanism that is as valuable as the content produced (Figure 1).

**Figure 1:**
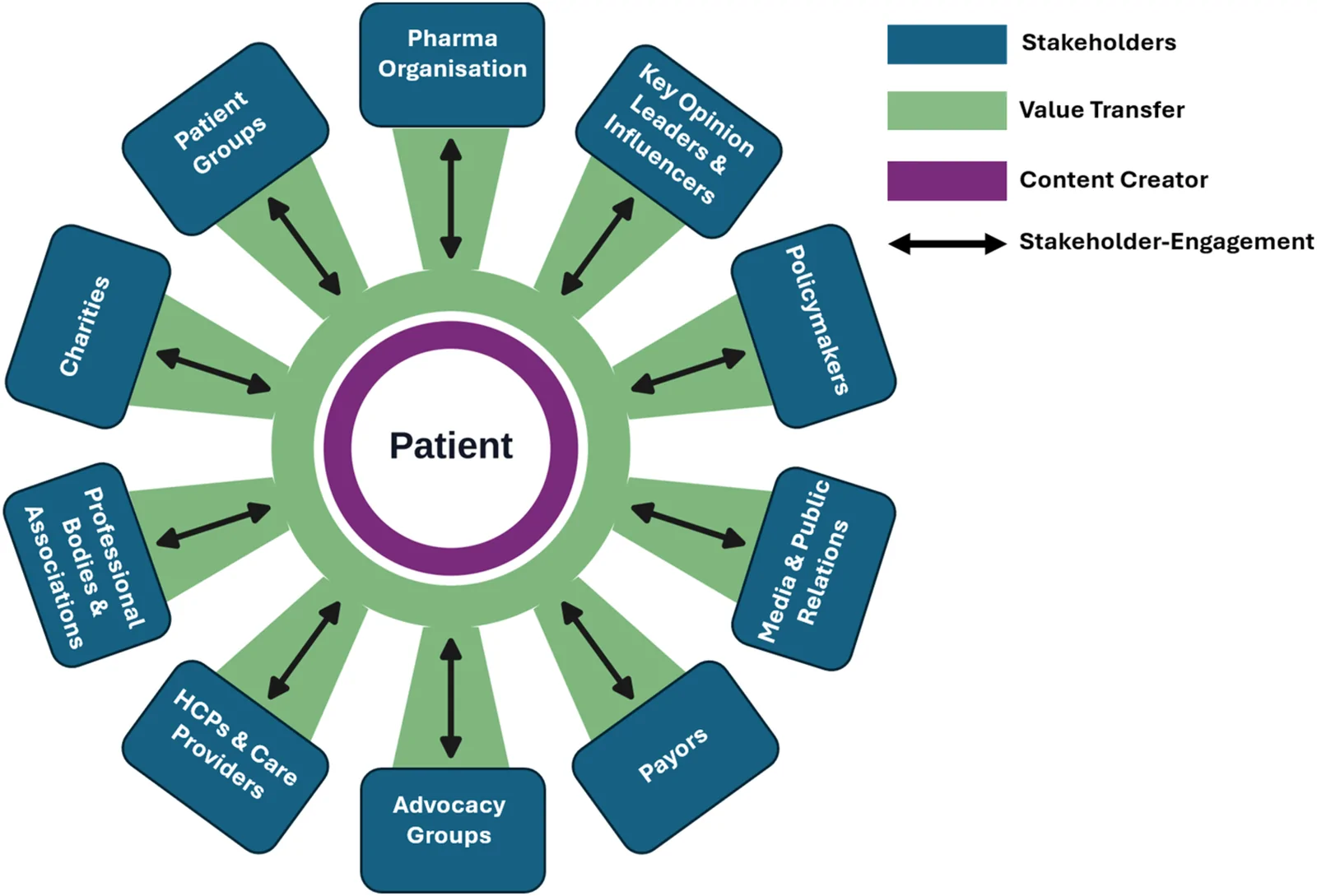
Authentic content serves as a mechanism for stakeholder-engagement. The content creator provides a mechanism for the patient to engage with different stakeholders without the patient having to directly “touch” each stakeholder and this ensures that all engagements are patient-centric and all stakeholders can “hear” the patient's voice. The patient, whose lived experience is being captured with or without involvement of other stakeholders like family members and caregivers, remains independent of the stakeholders and freed of influence and the stakeholders, often precluded from having direct relations with each other or restricted from it in some jurisdictions, are all participants in a mechanism that enables them to transparently collaborate without the need for physical and direct engagement—creating a shared value that benefits all stakeholders but which is especially captured by the pharma organisation.

Novartis UK's *Fighting Failure* campaign for Entresto (29) shows how authentic content creates a stakeholder-engagement mechanism that can drive structural change. 80% of heart failure diagnoses occurred post-emergency hospitalisation, representing a primary care failure (30) so Novartis devised a collaborative, multi-stakeholder campaign built on “powerful stories from heart failure patients” as part of a transparent co-creation mechanism comprising patient groups, professional bodies, charities and policymakers including the UK Health Secretary (31), and authentic content featuring patients and stakeholders that helped secure pledges for a system-level service redesign (32). Centred around a documentary called *Fighting Failure* about a heart failure patient, this mechanism enabled these to become co-creators, sponsors and catalysts for attracting other stakeholders. Initially hoped to be target audiences, these organisations became non-transactional stakeholders, collaborators and diffusion partners for content and messaging.

Even before the documentary was completed and broadcast with Amazon (33), its mechanism had already appeared to meet its goals and create value in ways conventional collaborations and conventional video content cannot. Campaign-linked evaluations estimated that the mechanism was associated with system redesign occurring 3–5 years faster (34) compared to industry norms for new treatment adoption, a compression of the “advocacy lag” by over 3 years (35), faster patient access of 3–6 months per patient (36), increased stakeholder-adoption (37) and system-wide diffusion within months rather than years (38). This £300,000 initiative generated an estimated ROI of between £11.9-£23.8 million annually (during 2021–2023) (39) and was credited with increased access and improved outcomes for NHS patients, although these figures derive from campaign-linked evaluations rather than independent controlled analysis and should be interpreted as indicative rather than causal.

Another benefit of featuring real people in content is advocacy: when patients, caregivers or other stakeholders contribute beyond initial participation, resulting in amplification of stories and messaging. In *Fixing Dad*, two caregiver sons created via Kickstarter content (40) about their diabetic father and their advocacy led to collaboration and co-creation with London Marathon, Prudential and the BBC (41), coverage of which led to deeper engagement with NHS stakeholders and extensive free/earned media coverage across television, radio, press and social media. Content initially seen by thousands led to advocacy and diffusions that reached many millions without any media investment.

### 2.3 Conceptual mapping of stakeholder-engagement and value-creation

The health innovation system is a complex process, takes many years and requires considerable investment and risk without a single timeline for how long solutions take to traverse through different stages. The principal stages are summarised in Table 1.

**Table 1:** Stages of health innovation system with corresponding functions (based upon pharma manufacturers and regulated providers).

Pre-Clinical Research/ Investigational New Drug Application	Clinical Trials	New Drug Application (NDA) Submission & Regulatory Review	Approval & Global Deployment	Local Market Deployment	HCP Engagement & Patient Access
2–7 years	6–10 years	1–2 years	1–2 years	Highly variable	Highly variable
Collaborate with academic institutions, research organizations and laboratories for funding, data sharing and early-stage validation	Recruitment of patients and stakeholders	Compile clinical trial data and submit NDA	NDA approval	Obtain local regulatory approvals for each market	Conduct medical education programs for HCPs
Acquisition, strategic alliances and IP protection	Phase 1–3 trials	Regulatory review of NDA	Establish manufacturing processes/quality control	Ensure compliance with local laws and regulations	Launch comms campaigns and engage with HCPs
	Reporting and presentation of results		Global market launch	Finalize budgeting, pricing and reimbursement negotiations	Develop patient support programs and educational materials
	Patient tracking and reporting		Regulatory submissions to individual countries	Establish distribution channels and partnerships	Ensure availability of the solution in pharmacies, hospitals and via relevant digital channels
			Pricing and reimbursement negotiations		Monitor/report efficacy, adverse events and drug safety, updating labelling
			Market access approvals and establishing distribution networks		Provide ongoing medical education for HCPs, patients and caregivers

The typical pharmaceutical development timeline involves multiple stages of development from early research to patient access. It begins with pre-clinical research and IND preparation (2–7 years), followed by clinical trials (6–10 years), the longest phase of development. Afterward, a New Drug Application is submitted for regulatory review (1–2 years), leading to approval and global deployment (1–2 years). The final stages, local market deployment and healthcare professional engagement and patient access, are highly variable and depend on regional regulations and market adoption.

According to OECD (42), “successful development of a new medicine takes an average of 10–15 years” and “the probability of obtaining marketing approval for drugs entering Phase I clinical trials ranges from 7% to 45%.” Early value-creation takes 8–17 years (43), involving complex stakeholder-engagement (44), intricate deployments towards HCPs and other stakeholders and, ultimately, patients within sophisticated regulatory frameworks. Historically, about *5%–8%* of drug candidates entering Phase I trials achieve launch in major markets (45), and, while 10% of drugs “work” scientifically, only 5%–6% complete full cycles of innovation to become globally available (46).

The HIS Framework (Figure 2) represents a new way of conceptually mapping the growth of stakeholder-engagement and value-creation across sequential stages of a drug's innovation lifecycle, positing that diffusion of “authentic content” increases collaboration, engagement, adoption and value-generation, and accelerates progression through stages. Authentic content has been created and deployed within the context of the HIS Framework in initiatives involving many global healthcare companies (including AstraZeneca, Roche, Boehringer Ingelheim, Novartis, Nestlé Health Science, Dexcom and others across 20 countries) as a model for embedding and communicating patients' voices but, until now, there has been little publicly-available evidence to support it. Without this evidence, these authentic content initiatives risk being seen as softer, qualitative and anecdotal “nice-to-haves” during later stages of innovation rather than concrete value-drivers that enhance the trajectory of innovation (47). Because some of these applications involved initiatives in which the author's organisation participated, those are presented here as illustrative practice-based observations rather than independent validation of the framework (see Conflicts of Interest and the Limitations section).

**Figure 2:**
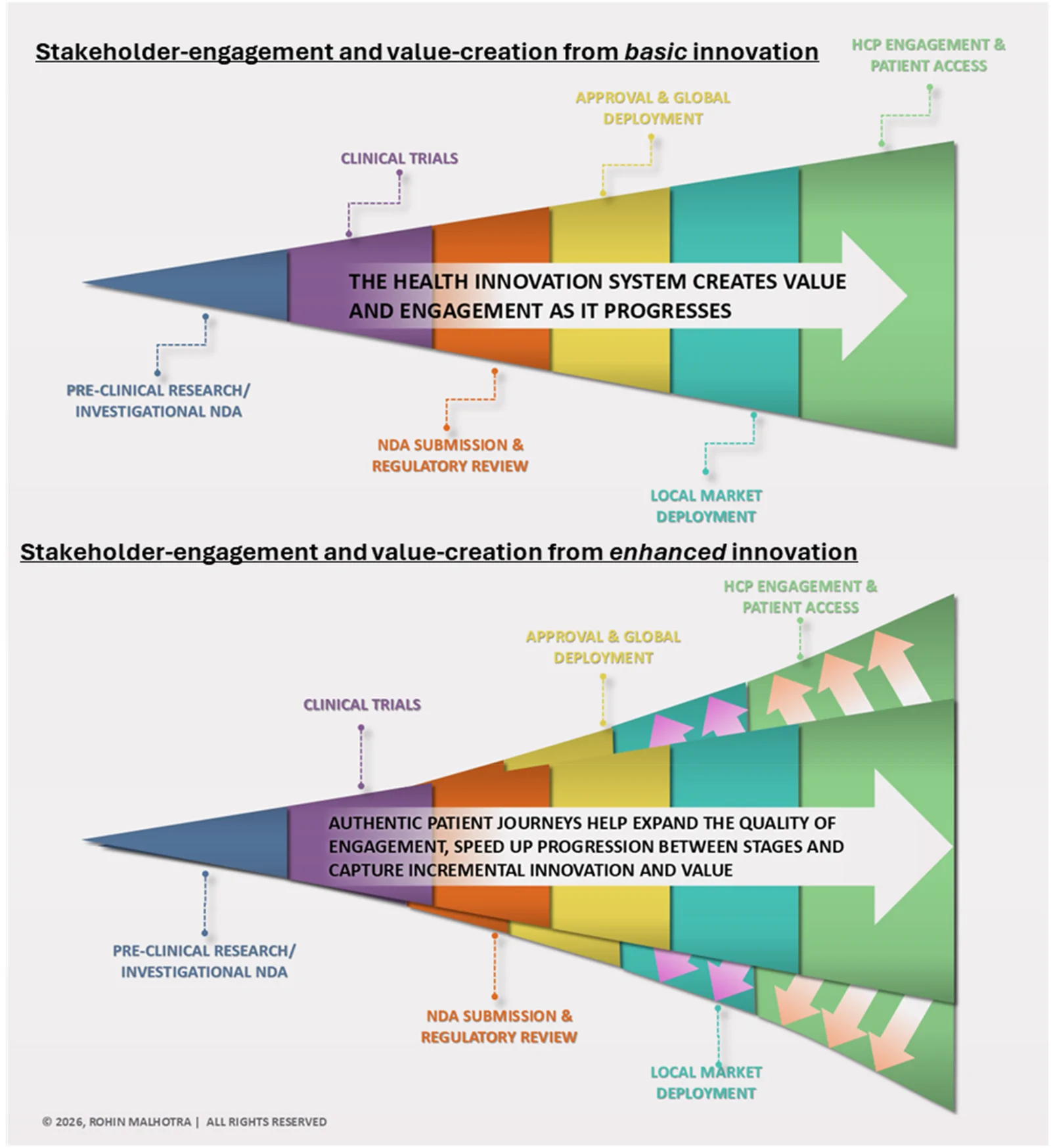
The health innovation system framework—stakeholder-engagement and value-creation from “basic” and “enhanced” innovation trajectories. Caption: The HIS Framework compares a basic innovation trajectory with an enhanced model in which authentic patient journeys and stronger stakeholder-engagement increase value creation across development stages. The basic trajectory shows steady gains in engagement and value and the enhanced trajectory illustrates the impact of broader collaboration, faster progression, and greater cumulative value-capture from diffusion of authentic content at different stages—highlighting how accelerated development and improved engagement helps maximize effective market exclusivity and generate wider benefits and market access.

The HIS Framework contrasts a “basic” innovation trajectory vs. an “enhanced” trajectory where authentic content amplifies stakeholder-engagement and value-generation and accelerates progression across stages. In the *basic* scenario, value-creation is relatively consistent whereas the *enhanced* scenario shows wider collaboration, faster stage progression and greater cumulative value-capture. The difference represents the hypothesised additional engagement and value, with acceleration that is proposed to yield multiplicative benefits.

Typical nominal patent lives for drugs are 20 years from initial filing date but effective commercial market exclusivity is usually shorter, averaging 7–12 years due to significant time before drugs are commercially marketable (48) so there are clear benefits from greater collaboration, enhanced engagement and accelerated progression through stages. Improved stakeholder-engagement for one drug can also yield synergistic benefits that go beyond that drug (49), including network effects, relationship spillover, enhanced reputation, tacit knowledge transfer, portfolio-level capabilities, talent acquisition, and operational and inter-organisational synergies.

The HIS Framework can also be expressed in conventional graph form (Figure 3), with the stages of the health innovation system on the horizontal axis and cumulative stakeholder-engagement and value-creation on the vertical axis. Presented this way, the basic trajectory rises steadily as innovation progresses, while the hypothesised enhanced trajectory diverges from it once authentic content is diffused (illustrated here from the Clinical Trials stage) with the gap between the two curves widening incrementally at each subsequent stage to represent cumulative, compounding engagement and value-capture. This convex, cumulative form is consistent with the growth curves described in diffusion of innovations research (50, 51), although the trajectories shown remain conceptual illustrations of the hypothesis rather than empirical estimates.

**Figure 3:**
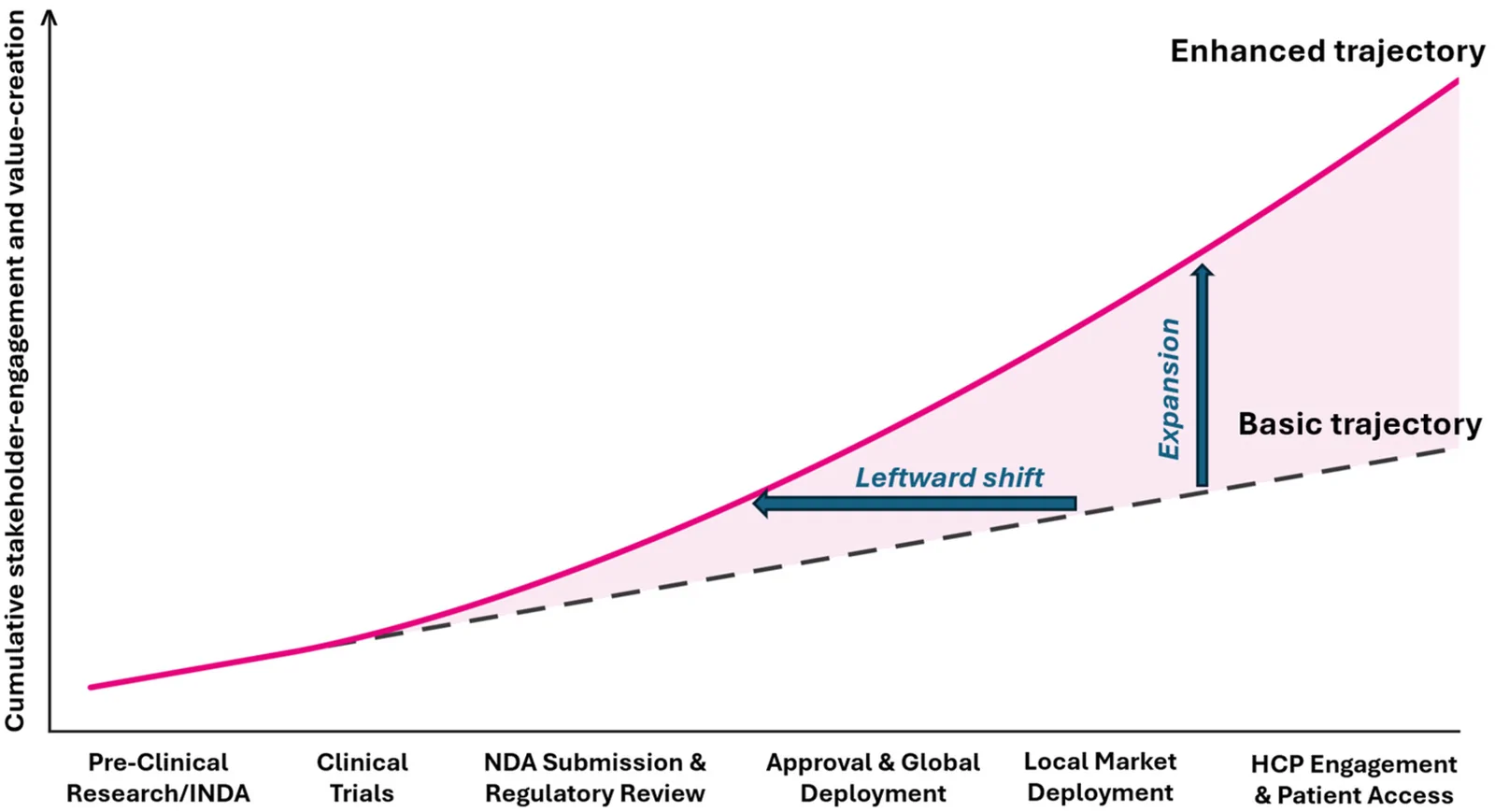
Stakeholder-engagement and value-creation from the HIS framework expressed as a graph. The basic and enhanced innovation trajectories of the HIS Framework are represented as cumulative curves. The basic trajectory (dashed) shows steady growth in stakeholder-engagement and value-creation across stages and the enhanced trajectory (solid) diverges once authentic content is utilised, with the shaded area representing the hypothesised cumulative difference in incremental engagement and value between the two trajectories. The curves are conceptual illustrations of the hypothesis and are not drawn from empirical data.

Despite these positive observations, measuring direct ROI of authentic content in pharma innovation remains challenging. Unlike industries with tighter feedback loops, pharma unfolds over long timelines and involves a “dense web” of stakeholders that define “success” and “value” differently amidst systematic impacts and complex interactions that obscures causality (52).

This complexity creates an attribution paradox, when benefits and “knock-on effects” are visible but difficult to attribute and, by the time benefits are realized, original contexts often vanish, complementary assets emerge and even failed programs generate knowledge or other benefits that improves future success rates without factoring into financial metrics. Most value is latent and co-created by multiple factors and evolving market conditions obscure simple *before-after* comparisons (52, 53) and traditional metrics may not capture gains from trust, engagement and company culture.

Effective measurement of patient-engagement impact is regarded to be an important goal (20) and this paper seeks to advance understanding by examining the hypothesis relating to how authentic content influences engagement, outcomes, innovation and ROI at each stage of the HIS Framework.

### 2.4 Theoretical foundations of the HIS framework

Although the HIS Framework emerged from applied practice, its propositions can be situated within, and are strengthened by, several established bodies of theory. Its central claim—that diffusion of authentic content expands stakeholder-engagement and accelerates progression across innovation stages—is most directly informed by diffusion of innovations theory (50, 51) which holds that adoption of innovations is driven not only by their objective merits but by how they are communicated through social systems over time. Authentic content maps closely onto several attributes known to predict adoption, notably observability (real patients visibly experiencing benefit), relative advantage (communicated in terms that matter to adopters) and compatibility with stakeholders' values, and the framework's “diffusion partners” correspond to the plethora of health innovation system stakeholders, opinion leaders and change agents identified as decisive in health sector diffusion (54).

The framework is equally interpretable through implementation science. The Consolidated Framework for Implementation Research (55) identifies evidence strength, stakeholder engagement and attention to patient needs as core determinants of successful implementation, and authentic content can be understood as acting simultaneously on several of its domains—the perceived evidence and relative advantage of the intervention, the “outer setting” (patient needs and external networks) and the “inner setting” (the culture and climate of implementing organisations). Implementation-outcome taxonomies (56) additionally supply validated constructs—acceptability, adoption, penetration and sustainability—through which the framework's engagement claims can be measured, as elaborated in the Discussion.

The mechanism proposed for why authentic content engages stakeholders draws on narrative medicine and narrative communication theory. Narrative medicine positions the absorption and interpretation of patients' stories as central to empathic and effective care (17, 57), while narrative communication research indicates that stories influence attitudes and behaviour through mechanisms—such as transportation, identification and reduced counter-arguing—that differ from those of didactic or statistical messaging (15, 16). Behavioural science supplies a complementary mechanism: social cognitive theory (58) predicts that observing “someone like me” succeed builds self-efficacy through vicarious learning, consistent with the peer-modelling effects reported later in this paper.

The framework's emphasis on co-creation also connects with design research and, in particular, service design, where co-production, co-creation, user involvement and empathy are well-developed analytical perspectives (59–61). Viewed through this lens, the content-creation process described in Figure 1 operates as a form of co-production: patients and stakeholders participate in designing the artefacts through which their experiences are communicated, rather than being passive subjects of them. Frameworks of participation offer a complementary analytical tool. Arnstein's “ladder of participation” (62) and its healthcare-specific development by Tritter and McCallum (63) distinguish tokenistic from substantive forms of involvement, and in doing so they sharpen an important boundary condition for the hypothesis advanced here: authentic content is proposed to function as a mechanism for embedding patient voices only insofar as participants genuinely shape the content and its diffusion. Deployments in which patients' narratives are captured but not permitted to influence decisions would sit low on the participation ladder and would not be expected to yield the engagement and value effects proposed by the HIS Framework.

Finally, the framework's emphasis on multi-stakeholder value-creation reflects stakeholder theory (58), which conceptualises organisational value as generated through networks of relationships rather than dyadic transactions, and contemporary stakeholder-engagement research in global health (49). Situating the HIS Framework within these literatures clarifies that it is not a wholly novel theoretical departure but an integrative, domain-specific application of established theory—and it identifies the constructs through which the framework can be operationalised and tested.

## 3 Supporting the hypothesis: how authentic content influences outcomes at different stages of innovation

A note on the evidence base is warranted before proceeding. The sections that follow draw on three tiers of evidence: (i) peer-reviewed empirical studies, including controlled evaluations and systematic reviews; (ii) industry analyses, case studies, reports and other grey literature, which are illustrative but were not conducted under controlled conditions; and (iii) practice-based observations, including initiatives connected to the author's organisation, which are the least independent form of evidence presented. Findings from tier (i) carry the greatest evidential weight whilst tiers (ii) and (iii) should be regarded as hypothesis-generating and -supporting rather than confirmatory, and the strongest claims in this paper should be read with these distinctions in mind.

### 3.1 Little evidence of authentic content's influence during nascent stages

There is little evidence of diffused authentic content within the nascent pre-clinical research and investigational NDA stages, which traditionally involve scientists and researchers working on molecular or technological advancements, although there is evidence to suggest it shifts the culture of research labs by connecting bench scientists with human realities of diseases they study, accelerating collaboration at earlier stages.

One pre-clinical study reported that having scientists watch videos with authentic accounts of patients describing daily challenges with a disease increased researchers' motivation and “purpose-driven” mindset in shaping research questions and adjusted experimental priorities to target patients priorities (64), and organisations also report that where loss of R&D personnel contributes to project delays, this authentic connection helps retain top scientific talent by reinforcing the meaningful impact of their work.

In rare diseases, early engagement via patient stories can be especially valuable. Patient advocacy groups often possess deep knowledge of disease natural history that may surpass what is published and so sharing authentic content comprising patient and caregiver experiences with pre-clinical project teams has led to identification of novel targets and biomarkers that might have been overlooked in the literature (65). Whilst more research is required in this arena, this underscores how, even before any clinical testing, patient voices can shape scientific direction and how shift investment upstream to drive “return-on-engagement” in R&D with the same rigor as some traditional R&D investments (66).

### 3.2 The potential role for authentic content across clinical trials phases

In the initial iteration of the HIS Framework, the scope for increasing stakeholder-engagement and accelerating progression in clinical trials appears to be limited. However, there is evidence to suggest that authentic content is not just relevant during this stage but that this could be where its diffusion has the greatest potential impact.

The following section reviews evidence suggesting that authentic content may improve multiple facets of clinical trials, including study design, patient recruitment, activation of caregivers, conversion of inbound interest, and productive engagement with HCPs, PAGs, communities and charities (fostering partnership cultures and enhancing education, enrolment, retention, representation and diversity) as well as adding new dimensions and efficiencies to reporting results, bringing to life trials and highlighting impacts that go beyond just data for internal and external stakeholders.

One major challenge in trials is high operational failure rates, with 50% of investigative trial sites failing to enroll more than one patient (20) and about 80% of trials experience delays or closure due to insufficient enrollment (43). This is often not due to the molecule's efficacy but a failure of logistical empathy. Protocols designed for “ideal” patients and scenarios can lead to operational requirements that are incompatible with real-world lives and authentic patient stories can highlight these disconnects. When trial planners engaged actual patients to simulate study visits and provide feedback, patients identified burdensome requirements (like excessive clinic visits or rigid fasting schedules) that would deter participation.

In one case, 24 recommendations elicited from authentic patient simulations were adopted to simplify a trial protocol, resulting in the study finishing ahead of schedule and saving an estimated $1 million in costs (67), and an earlier cardio-metabolic trial that embedded patient-designed educational materials and included patient testimonial videos filled its enrollment 4.2 months faster than projected (750 days vs. 852 days) and increased completion from 81% to 92%, saving $1.1 million in potential dropout-related costs (68).

There is evidence to support how authentic content improves patient recruitment by building trust and relatability. Traditional recruitment materials are often text-heavy and impersonal, whereas authentic content like patient testimonial videos and first-person stories can “bring to life” the trial's importance and address fears. Trials employing patient storytelling in recruitment experience substantial boosts. Economist Intelligence Unit analysed 4,000 trials and found patient-centric trials (using patient insights and content in design) were 19%–21% more likely to launch successfully than traditional trials (69) and another study found it boosted recruitment and retention rates by up to 30% (57). As 85% of trials fail to retain enough patients to meet endpoints (3), these associations are potentially significant and may help avoid costly trial failures or extensions.

Authentic content can also broaden the pool of participants by reaching under-represented populations. By featuring diverse patient voices, content can resonate with communities that might distrust medical research and this has been linked to improved enrollment of minorities and broader demographic representation in some trials (though exact figures vary by context). An important contributor is trust—hearing “someone like me” describe their positive trial experience can overcome hesitation and, indeed, a recent survey found 86.8% of oncology patients say it is important or very important to speak with other patients like them before joining a trial (70) and, in scenarios where direct conversation is not feasible, authentic video testimonials serve as a proxy that provides peer reassurance, building trust and participation.

Another benefit is enhanced engagement with other stakeholders supporting trials, such as clinicians and trial site staff. When investigators and study coordinators view patient journey videos, it can reinforce the “why” behind the study and motivate them to recruit actively and keep patients engaged. An Applied Clinical Trials survey that compiled 650 engagement metrics from 22 sponsors observed that even *low-cost storytelling activities* (like lay-friendly trial summaries or online forums where patients shared experiences) led to faster trial startups, higher retention and reduced delays (20), in addition to “facilitating cultural change” by institutionalising patient voices to help employees become more committed and aligned to patient-centred goals (23, 27).

A 2020 study of “community engagement studios” also found patient stories play key roles in aligning research priorities with external community needs, establishing context and mutual understanding—“leading to more patient-centered innovations in clinical practice” (27) and “clinical trials with greater feasibility, enhanced recruitment and retention, and more relevant outcomes.” (71) Authentic content built empathy and shared purpose across researchers, clinicians, industry, regulators and patients (20, 27), reinforcing an earlier 2017 industry assessment of 121 patient-centric initiatives that linked “high-impact” storytelling to better engagement metrics and “faster study planning, approval and initiation timelines, higher randomization, recruitment and retention rates, fewer disruptions and delays, and more positive study volunteer satisfaction levels.” (71)

Modeling by the Tufts Center for the Study of Drug Development (CSDD) suggests that even small patient-focused changes may substantially shorten timelines, with material implications for ROI. A modest $50,000 investment (input to avoid a trial amendment using patient content) could yield an estimated +$25–32 million NPV by speeding up Phase II/III programs (72) and eliminating just one protocol amendment saves about 90 days through early patient input (73) and studies with patient organisations onboard tend to start and complete sooner due to more feasible protocol designs and lower attrition (71).

Even greater economic leverage can be achieved in earliest stages, where costs of pivoting are lowest and strategic trajectory is set. Levitan provides an example on quantifying patient-engagement value from authentic content using an ENPV model to simulate the financial impact of patient-centric initiatives—positing that relatively small investments yield disproportionate returns by affecting three key levers: avoiding protocol amendments, accelerating enrolment, and improving retention. The modelled financial impacts of these patient-engagement interventions are summarised in Table 2.

**Table 2:** Financial impact of patient engagement on oncology development programs.

Metric	Impact of Patient Engagement (Pre-Phase 2)	Impact of Patient Engagement (Pre-Phase 3)
Increase in Net Present Value	$62 million	$65 million
Increase in Expected NPV (ENPV)	$35 million	$75 million
Launch Acceleration	2.5 years	1.5 years
ROI on $100k Investment	> 500-fold	> 500-fold

Levitan, Therapeutic Innovation & Regulatory Science (2018) (73).

The financial and operational benefits of integrating patient-engagement early in the drug development lifecycle, specifically comparing impacts at the Pre-Phase 2 and Pre-Phase 3 stages, are significant. The table demonstrates substantial increases in Net Present Value (NPV) and Expected NPV (ENPV), with ENPV more than doubling from $35 million to $75 million when engagement is sustained into Pre-Phase 3, significant launch acceleration, ranging from 1.5 to 2.5 years, and a profound ROI exceeding 500-fold for a baseline $100,000 investment.

A single substantial amendment can cost $500,000 in direct costs and delay a trial by 60 days (74) so avoiding just one substantial amendment contributes significantly to the NPV increase. Authentic content that identifies barriers before trials start improves retention by 30% (75) and contributes significantly to a $62 m–$75 m increase in asset value for pre-Phase II/III projects (74). Levitan indicated investing in this “soft” science of patient listening yields “hard” financial returns, with a $100,000 investment in robust patient-engagement that prevents just one protocol amendment and improves retention increasing ENPV of a pre-Phase III asset by $65 million (73)—deriving value from getting therapies to patients sooner and maximising patent-exclusivity periods. Trials designed to fit into patients' lives, informed by authentic journey mapping and authentic content, helps patients understand treatments.

Replacing patients in Phase III is exponentially costlier than retaining them and authentic content helps improve retention, preserves statistical robustness and reduces needs to over-recruit (76), and data from CSDD and IQVIA (45) corroborates this. Patient-centric trial designs have been associated with cycle times reduced by up to 17% (77) and patient access accelerated by up to 3 years (78)—with not just faster trials but more robust evidence that reduces risk of catastrophic financial events (79) like regulatory rejection, restricted labelling or poor payer assessments. In Levitan's model, early patient-engagement accelerated launches by up to 2.5 years in Phase II and 1.5 years in Phase III programs, with the model attributing a large portion of NPV gain during peak-revenue years before patent expiration (73) that leads to ROI exceeding 500-fold (72).

In summary, the evidence reviewed suggests that authentic content may play a substantial role during clinical trials, being associated with more feasible, de-risked and patient-friendly protocols, shorter development timelines and higher enrollment, completion rates, trust and collaboration among participants, sites and sponsors. Indeed, the paper may support revisiting the shape of the HIS Framework so the *enhanced trajectory* begins to take effect earlier, resulting in earlier and broader stakeholder-engagement and value-creation. This positions authentic content less as a peripheral, qualitative tactic and more as a potential strategic component of trial-optimisation, and engaging patients with it “early and often” appears to be associated with greater feasibility, faster recruitment, better retention and more relevant outcomes for trials (71).

### 3.3 An effective way to embed patient voices into submissions and regulatory reviews

Progression from successful clinical trials to market access is perilous, and authentic content can help navigate payer negotiations and Health Technology Assessment (HTA) bodies to reduce the “valley of death” between approval and access (80). Regulatory agencies have launched patient-focused drug development initiatives to systematically include the patient voice in decisions (81) as these inputs help regulators understand real-world impacts of treatments beyond clinical endpoints. Authentic content can bolster submissions by highlighting unmet needs and outcomes, drawing attention to “invisible” burdens and serving as qualitative contextualized evidence on what matters to patients (82).

FDA's patient-focused drug development guidance explicitly encourages integrating patient perspectives in applications (69) and an EMA therapeutic area head noted that hearing directly from patients can “put a face” to the benefit-risk profile, sometimes tipping the scales in close decisions (83). HIV activism has expedited FDA approvals and enabled 30 HIV treatments to reach patients faster via surrogate endpoints (84) and, for rare diseases, similar initiatives proved instrumental for Duchenne muscular dystrophy advocacy, pushing FDA to approve *eteplirsen* earlier to fasten patient access (85).

HTA bodies evaluate whether new therapies provide sufficient value to justify reimbursement but traditional HTA metrics like Cost-Utility Analysis and Quality-Adjusted Life Year (QALY) are criticized as reductionist (86) as they often fail to capture nuanced, multidimensional impacts for rare, chronic or debilitating conditions and place disproportionately adverse burdens on caregivers (86). Authentic content can fill these gaps and a study of NICE submissions for highly specialised therapies found patient input (in the form of narratives or authentic content) provides “new evidence” or helps “interpret other evidence” in 100% of cases reviewed (87).

There are many high-profile case studies to support this. Cystic fibrosis drug *Orkambi* initially faced rejection by HTA bodies due to its high cost and uncertain long-term data but the cystic fibrosis patient community launched an “Action for Access” campaign featuring longitudinal patient content about the everyday “burden of illness” that patients and families publicly shared and, under mounting public pressure, this helped provide real-world evidence that highlighted how clinical trial endpoints did not fully capture the reduction in exacerbations and ability to return to work/school. Initial “no” decisions were overturned in multiple countries, leading to managed access agreements that made Orkambi available years earlier than under normal processes (88).

For Biogen's nusinersen, economic evaluations indicated between €400,000 and over €6 million per QALY, which were far above standard thresholds. However, patient groups successfully communicated values of “stabilization” via a campaign including video testimonies of families and patients (89), communicating daily functioning that QALY models undervalued, and this helped several HTA bodies to approve nusinersen, often with novel funding arrangements.

Despite standard reimbursement timelines of over 600 days, regulators and payers may expedite their processes when convinced of this real-world relevance. Pathways explicitly integrating patient values can reduce the 2-month gap between marketing authorisation and the publication of final guidance (90) and direct patient-engagement with Canadian HTA bodies increased developing equity-focused recommendations by nearly four-fold (3.85 “odds ratio”) (91).

In summary, for Regulatory and HTA submissions, authentic content serves as an applied form of visual ethnography and a persuasive narrative tool. It humanizes reviews and helps ensure decisions are not made on abstract data alone but with fuller understanding of patient impact—and it has been associated with faster approvals, more favorable reimbursement outcomes and creative solutions that can get therapies to patients sooner. Regulators “increasingly expect the authentic patient voice” as part of submissions and including authentic content can strengthen a case for approval (92) as a key part of evidence that determines access to innovation.

### 3.4 Driving multiple outcomes across approvals, global deployment and local market deployment

Once products are approved by regulators, the next challenge is global deployment and local market adoption, converting validated products into widely accessible ones. This involves scaling-up manufacturing, securing reimbursement, launching in different countries, educating HCPs on new therapies, integrating therapies into clinical practice guidelines, organising supply chains. and driving uptake among providers and patients.

There is a risk that the “patient” becomes an abstract concept in budgeting or logistical discussions, and authentic content can play a vital role in smoothing this rollout and expediting access across markets—serving as potent reminders to keep patient outcomes at the center of deployment plans. A video of a real patient describing how a new treatment changed their life can have more influence on committees than dossiers of statistics, especially in areas of high unmet need (92).

Patient narratives can feed into global planning by emotively communicating patient needs that align stakeholders with urgency and, locally, patient stories can generate grassroots demand and trust for new therapies. When authentic content is shared (through presentations, events, community forums, conferences, mainstream media, social media), it raises awareness and enthuses key opinion leaders (KOLs), patient groups, clinicians, hospitals, policymakers, local markets and wider populations.

Novartis' *Fighting Failure* initiative is a good example of this, using authentic content to build a coalition of support that created pressure for accelerated systemic changes, and there is also a successful clinical adoption of dupilumab for atopic dermatitis, which was supported by the integration of Patient-Reported Outcomes (PROs). While standard clinical efficacy metrics drive initial formulary inclusion, its reporting suggests that real-world patient narratives and qualitative data detailing improvements in quality of life are crucial for shifting clinical perception, guiding shared decision-making, and driving provider utilisation (93).

It is important to note that much of the deployment phase is internal within companies so employee buy-in, cross-functional coordination and adoption by local affiliates are key. Authentic content engages external stakeholders but also energizes internal stakeholders, health systems and organizations integrating patient stories into their culture, helping improve performance indicators—although much of the supporting evidence is constrained by confidentiality or, if diffusable (often because it has been successful to some degree) is observational, anecdotal, qualitative, or case-based, resulting in biased reporting towards favourable outcomes unless conducted with public bodies with a duty for disclosure regardless of research outcomes. The US Agency for Healthcare Research and Quality (AHRQ) reports that collecting and acting on patient narratives can “catalyze staff improvement and bolster [patient satisfaction] survey scores” (94) as these patient journeys and care pathways often uncover systemic gaps and inspire people to drive changes that improve outcomes.

A US study found that hospital staff who frequently heard patient stories had higher overall patient experience ratings in their units (22) and, in pharma companies, high employee engagement correlates with improved care processes and clinical outcomes in the partnered health systems (94). When innovations are co-created with patient voices, stakeholders report faster and broader adoption, and this may meaningfully influence internal culture, potentially helping address critical issues of burnout and talent retention. Healthcare industries face high burnout rates and connecting employees to the purpose of their work is a potent tool for retention and emboldening people. Purpose-driven engagement initiatives have been reported to bolster employee resilience and deepen organisational empathy, with implications for workforce stability (95) and this “purpose premium” may reduce recruitment costs and improve the quality of workforces (96).

Takeda's “In Their Shoes” US campaign involved employees simulating living with Inflammatory Bowel Disease (IBD) (96) and measured significant improvements in prosocial job perceptions (97), empathic connection, organisational innovation (98), higher morale and “emboldenment” of staff to propose new initiatives (99). Employees who felt greater satisfaction and renewed connection to patients were less likely to leave. Authentic content helps facilitate a visceral connection that slide presentations and static reports cannot deliver (97), demonstrated by another global pharma company that used a 3 min patient film for an internal presentation, which accelerated multi-country deployment when only one pilot market was initially expected (100).

A broader implication is that authentic content can drive education, morale and organisational culture, embedding authentic content into environments to help engagement, collaboration and innovation thrive. Replacing specialised life sciences employees is costly, with estimates ranging up to several multiples of annual salary in industry analyses, and a BioSpace survey indicated that 67% would look for a new job within 12 months (101), with “meaningful work” cited as a top attribute for ideal employers (95). Connecting employees to real-life patient experiences may serve as a purpose-driven retention mechanism and hearing a patient story makes clinical education more “realistic” and significantly improves student attitudes for all core competencies (communication, roles, teamwork and ethics) (102).

Actively sharing patient stories with employees has been associated with positive effects on motivation and cross-functional teamwork, and with cultural shifts toward patient-centricity. In one PharmaVoice account (103) a gastroparesis patient advocate and her husband spoke to pharma employees and reported a “strong impact on those in attendance” as departments gained deeper appreciation of their reality and, in another Servier case study, an “interactive gallery” of filmed patient experiences not only generated actionable ideas (84 project ideas were crowdsourced across departments in an internal “patient-engagement challenge”) but catalysed collective shifts from a traditional, siloed approach to a more collaborative, patient-centered model (104).

When local markets prepare to operationalise new treatments, many initially lack turnkey readiness required by global strategies. Despite existing infrastructure and expertise, deployment often requires reassigning personnel from unrelated therapeutic areas, new talent and relationships and disease-specific knowledge (105). Authentic content embeds patients' voices and supports: faster buy-in from local stakeholders; builds trust with providers and payers; creates momentum for adoption; humanises innovation; helps bridge gaps in understanding; supports more consistent implementation across health systems; improves the pace and quality of local market adoption by enhancing emotional resonance; and fosters alignment and local collaborations that support compliance efforts, strengthen HCP engagement and provide mechanisms that can accelerate local activation.

In summary, during the approvals and these deployment stages, authentic content functions on two levels: externally, it educates and motivates healthcare systems and their stakeholders to adopt innovation, and internally, it aligns, engages and energises people and organisations. Addressing scepticism with authenticity emboldens and forms coalitions of support from closer proximity to patient voices and authentic experiences that increase value-capture and help patients gain access faster.

### 3.5 An established driver of outcomes across HCP engagement & patient access

Once new innovations are accessible by markets, the focus shifts to uptake, adherence and long-term patient outcomes. This stage involves engaging front-line HCPs to prescribe or recommend solutions, and supporting patients to adhere and benefit from them. Authentic content can play a valuable role here by facilitating education, trust, and behavioural change among providers and patients, and the HIS Framework shows how effective engagement expands collaboration and value-capture, and how markets benefit exponentially from efforts to accelerate progression made during earlier stages.

The scope of activities available to support HCP engagement and patient access varies significantly across jurisdictions, where some permit direct patient communications but others impose stringent restrictions to protect patients and permit limited messaging to HCPs, but a consistent challenge is to achieve effective and broader stakeholder-engagement (100). Indeed, content featuring patients is frequently designed for other stakeholders only. A 2022 review of transitional care programs for chronically ill adults found caregiver engagement significantly reduced hospital readmissions by 11% at two or more months post-discharge (106), supporting the premise that engaging non-patient stakeholders also improves outcomes

Many organisations maintain more traditional pharma commercialisation techniques that rely on *share of voice* and flood markets with data-heavy materials and conventionally curated promotional and brand-centric communications. However, HCPs are increasingly resistant to this, with 68% of HCPs reporting feeling occupational and emotional exhaustion (107), and so authentic storytelling offers an alternative that taps into emotional and intuitive cognition (sometimes referred to as “System 1” thinkin (108)) and, by telling real patient stories, companies can capture HCPs' attentions in ways glossy brochures cannot. One study found that pharma video marketing (featuring patient or physician stories) generated ROI of about $60 for every $1 spent, roughly double ROI of traditional email campaigns (109), and a systematic review and meta-analysis found visual-based interventions significantly improved health literacy outcomes compared with text-based formats (110), and a more recent analysis of a migraine medication campaign featuring patient and HCP narratives demonstrated increases of 29% of patients starting the therapy and 19% of HCPs prescribing it (111) (suggesting an association between “resonance” and volume).

Further evidence relating to authentic, personalised engagement lies in medication adherence, with longitudinal analysis of AbbVie's Humira PSP, which utilised “Nurse Ambassadors” and personalised engagement (112) and achieved 29.3% higher adherence compared to matched non-PSP patients and a 22% lower discontinuation rate with PSP patients. Patients stayed on therapies longer, revenue increased 12.2% higher and this was offset by a 35% reduction in disease-related medical costs and 9% lower PSP healthcare costs (113).

In pharmacies, comparisons between traditional retail and high-touch Specialty Pharmacy Providers (SPPs) using authentic content, narrative-based counselling and closer patient relationships for a multiple sclerosis injectables resulted in 58% of patients using the SPP remaining on therapy after one year, vs. only 37% for retail, and annual incremental revenue per patient in the SPP was $8,717 higher for one product and $9,055 higher for another product and, for a 2,000 patients cohort, this authentic engagement captured over $17 million annual value (114).

Underpinning these outcomes is the issue of trust, which is fundamental to behavior change. The 2025 Edelman Trust Barometer shows trust in pharma (59%) lags behind trust in “my doctor” (79%) and “my pharmacist” (73%) (115), and trust is becoming decentralized with 64% of people saying “someone with lived experience” is a credible source of health information (115). This suggests pharma would benefit from revisiting how it communicates messaging, and how authentic voices (with patients, HCPs and stakeholders) can improve resonance and trust, and become partners in design rather than just subjects of study. This co-creation can accelerate innovation and increase value and outcomes on a global scale (116), using tools like authentic content and experience-based co-design increase trust (117) whilst ensuring it is not too “complex” for it to effectively engage (100).

When patients see others embarking on journeys to avoid similar jeopardies, it strengthens relatability, propensity for effective engagement, sharing and changed behaviours (118). Authentic content can be akin to “behavioral modelling” with patients seeing “someone like them” successfully engaging more likely to persist with therapy—including hypertension patients who watched videos of peers achieving blood pressure control improving hypertension management vs. those who received standard information (119) and diabetic patients who improved their glycemic control after viewing peer narratives (120). This “If they can do it, so can I” effect (121) helps translate complex clinical instructions into relatable narratives and bridges the gap between “prescribed” and “taken.” (122)

Authentic content is more than just “soft” communications or a training tool; it has been associated with changes in care. A review of 64 studies found patient stories improve clinicians' empathy scores, clinical decisions and behaviours in 36 % of interventions (123), another study found video-narrative interventions increased self-efficacy for post-stroke medication adherence by +1.2 *standard deviation* and cut non-adherence 30 % (124) and another systematic review showed digital patient stories boosted “shared decision-making” metrics by +15 *points* on average (97). Clinics whose staff frequently shared patient narratives recorded significantly higher patient experience scores (22) and, when “unbranded content” featuring three patient journeys was deployed to physicians by pharma sales teams for a cardiovascular drug, meetings between them increased by 260% after one month and by nearly 600% after 6 months (125)—with physicians more willing to meet when discussions centered on real patient experiences rather than pure product features.

The impact of authentic content across broader consumer channels was also evident during Dexcom-sponsored broadcasts of FIXING US, a NBC reality-series in the US featuring patients using G6 continuous glucose monitoring devices (126). Broadcasts generated an 89% uplift on personal and wellness decisions (127) and a 94% uplift on likelihood to research or seek out more information (127) but the authentic content also significantly increased brand-related metrics—challenging perceptions that authentic content is more aligned with non-branded activity (128). Even for Dexcom's G6, which had high public awareness and market share (129), authentic content was associated with a 37% increase in brand awareness, a 120% increase in intent to use the brand and a 77% increase in favourability to recommend the brand (127).

In summary, the evidence reviewed suggests authentic content can function as a high-value intervention with HCPs and patients, helping innovations to tackle clinician fatigue, motivate education and build resonance and adherence with patients by leveraging peer influence and humanising treatment experiences. It fosters trust in the face of continued erosion of trust in traditional advertising or top-down communications, suggesting that it may be more than a “soft” communications effort, with measurable associations with collaboration, health outcomes and financial results.

## 4 Discussion

### 4.1 The potential impact of earlier deployment

There is evidence to suggest that the earlier authentic content is deployed in innovation lifecycles, the greater its potential impact. While embedding patients' voices is increasingly recognised as an important contributor to innovation design, deployment and adoption, research suggests its timing may be equally critical.

Early-stage inclusion of authentic content diffusions not only improves relevance, progression and design quality but also appears to activate a more profound shift in stakeholder alignment, innovation culture, and system-wide engagement. When diffused early, authentic content may prime the system by shaping expectations, mobilizing trust, and clarifying unmet needs, potentially allowing subsequent stages of design, development, increasing adoption (50) and deployment to proceed with greater alignment and efficiency vs. later-stage deployment (51).

Earlier deployment can be interpreted as not only affecting the speed of progression but the setting of innovation trajectory. Decisions made during early stages of research and clinical development tend to become path-dependent, influencing protocol design, endpoint selection, stakeholder expectations and evidence strategies that are increasingly difficult and costly to modify later. In this context, authentic content functions less as a downstream communication asset and more as an upstream system input that helps define priorities before organisational and regulatory inertia become embedded, and authentic content can reduce the need for later corrective adaptations—thereby improving feasibility and coherence across subsequent stages of development.

A further implication is how value generated through earlier deployment has cumulative, as opposed to simply linear, effect. Early improvements can enhance trial feasibility, recruitment and retention, which in turn strengthen datasets, facilitate regulatory dialogue and enhance reimbursement narratives, ultimately accelerating adoption and increasing effective periods of market value-capture. This suggests that benefits observed at later stages may partially represent compounding effects initiated upstream, where relatively small interventions produce disproportionate downstream benefits—dynamics that align with economic models indicating that early patient-engagement yields asymmetrically higher returns because the cost of strategic pivoting increases substantially as development progresses.

It is also useful to consider that the impact of enhanced culture and trust may be deeper, in terms of how it reduces resistance between commercial, medical, regulatory and access teams, globally and locally, and how it improves the propensity for innovation. It can be surmised that this does not just improve individual initiatives and stages but potentially results in greater receptivity for contributions and a more collaborative and fertile environment for generation of ideas.

This less-siloed decision-making and improved cross-functional collaboration also activates broader stakeholder networks earlier in the innovation lifecycle and provides a plausible explanation for the observation that early authentic content diffusions can generate knock-on effects beyond immediate purposes of diffusions like systemic risk-reduction, which can increase the likelihood of successes and viability of innovations that might otherwise struggle to succeed without an enhanced health innovation system within which it can thrive.

Finally, there is also a case for redesigning the HIS Framework, shifting initial expansion of stakeholder-engagement and value-generation leftwards to the start of the Clinical Trials stage, leading to increased latent value-creation as a result of an earlier enhanced trajectory. Several studies have linked early patient-engagement in Clinical Trials to faster recruitment, better adherence, and reduced downstream costs across all Phases (79), and authentic content may be not only relevant during this stage but potentially at its most impactful (130). This proposed earlier trajectory is illustrated in Figure 4.

**Figure 4:**
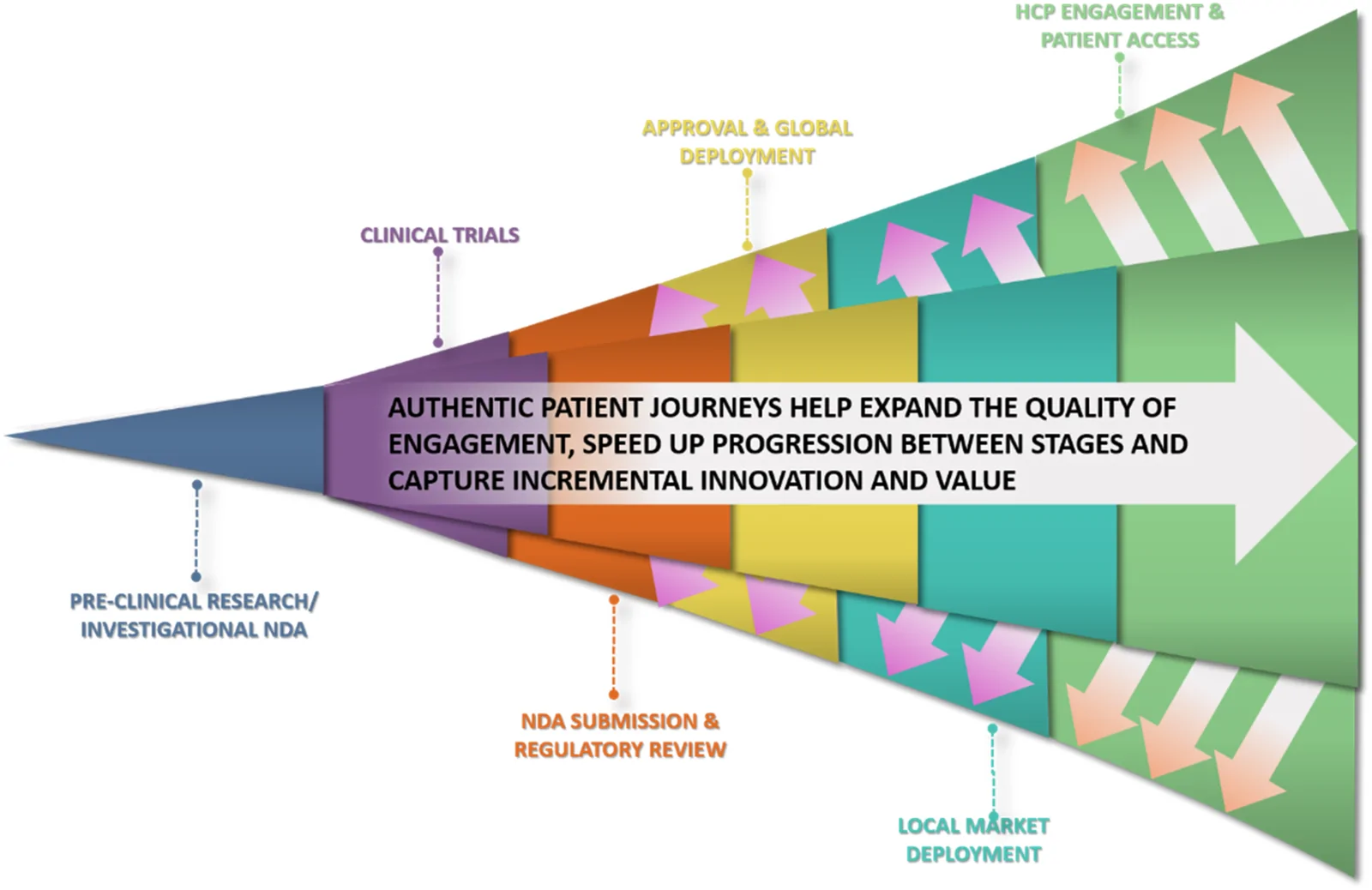
The “Redesigned” health innovation system framework—earlier “enhanced” innovation trajectory. The “Redesigned” HIS Framework illustrates an earlier commencement for the enhanced trajectory for stronger stakeholder-engagement and increased value-creation across development stages as a result of evidence relating to how authentic content can help lead to broader collaboration, faster progression, and greater and more prolonged cumulative value-capture from diffusion of authentic content at different stages.

While further empirical work is needed to quantify these effects, evidence presented here aligns with existing research suggesting that authentic content may deliver its highest value when it shapes trajectory, creates a more fertile innovation system and speeds the pace of progression of development. Rather than being viewed primarily as a mechanism that amplifies value later in development, evidence suggests its greatest potential impact may occur when stakeholder-engagement expansion begins earlier, effectively shifting the enhanced trajectory leftwards toward the commencement of clinical development. While further empirical work is required to quantify the magnitude of these effects, the emerging pattern indicates that timing may be a critical determinant of value generation, shaping not only the pace but also the direction, resilience and cumulative impact of innovation pathways.

The findings suggest authentic content may be more than just a *soft* communications tool and more akin to a strategic upstream imperative and system input that defines the innovation trajectory, stakeholder cognition, and subsequent decision-path dependencies, especially the earlier it is diffused.

Integrating patient voices before organisational and regulatory inertia takes hold is hypothesised to result in a “leftward shift” in the HIS Framework, and earlier deployment may have a cumulative and multiplicative effect—potentially accelerating progression of development from an earlier stage to a considerably longer period of effective commercial market exclusivity, enabling more patients to access medicines faster, and enabling providers to offer more efficient access as a result of greater scalability. The timing of authentic content deployment may therefore be a critical determinant of value, shaping the trajectory, pace, resilience, and long-term viability of the healthcare innovation pathway.

### 4.2 Leveraging artificial intelligence to scale authentic content integration

Despite benefits of deploying authentic content across health innovation system stages, extracting and scaling insights from vast qualitative data remains challenging but artificial intelligence (AI) and machine learning technologies present significant opportunities—although, as a nascent application of a new and evolving technology, this is a less rigorously researched area.

Whilst AI has revolutionised quantitative aspects of pharma (131), applying AI and Large Language Models (LLMs) to qualitative interview transcripts, video testimonies and other forms of authentic content has been slower due to complexities and resistance to accessing patients' data even when collected and accessed in accordance with compliance and legal rules (132). This stems from ethical dilemmas, trust issues, institutionalised hesitancy, regulatory ambiguity, data-management fears, training bias and implementation hurdles that are challenging for nuanced, human-centric qualitative data (132) but this is changing (50).

LLMs offer new ways to systematically analyse authentic content at unprecedented scale, able to sift through thousands of patient interviews to identify common pain points, unmet needs and other patterns. It also helps match patient stories to relevant decision-makers, ensuring the right story reaches the right stakeholders at the right time in ways both personalised and scalable.

Even if authentic content is created for specific targeted deployments, AI offers ways to extract insights from its footage to support planning and decision-making across all innovation stages from R&D and patient care (133). The ability to access AI-powered responses to prompts provided to repositories of “patient voices” and authentic experiences an almost unlimited number of times could substantially change how decision-making is supported and how time and money are saved. Delaying decisions to elicit research is costly and constrains innovation, with knock-on effects for subsequent rollouts that costs millions and demoralise stakeholders, but AI-powered tools may help avoid this and generate substantial value.

One development in this arena is *Authentic Intelligence*, a generative-AI LLM devised in 2024 and launched in 2025 by FIXING US (the author's organisation) with US software provider, NomicAI (134) (an example included for illustration only and thus subject to the interpretive-bias limitations discussed below). The user interface is shown in Figure 5.

**Figure 5:**
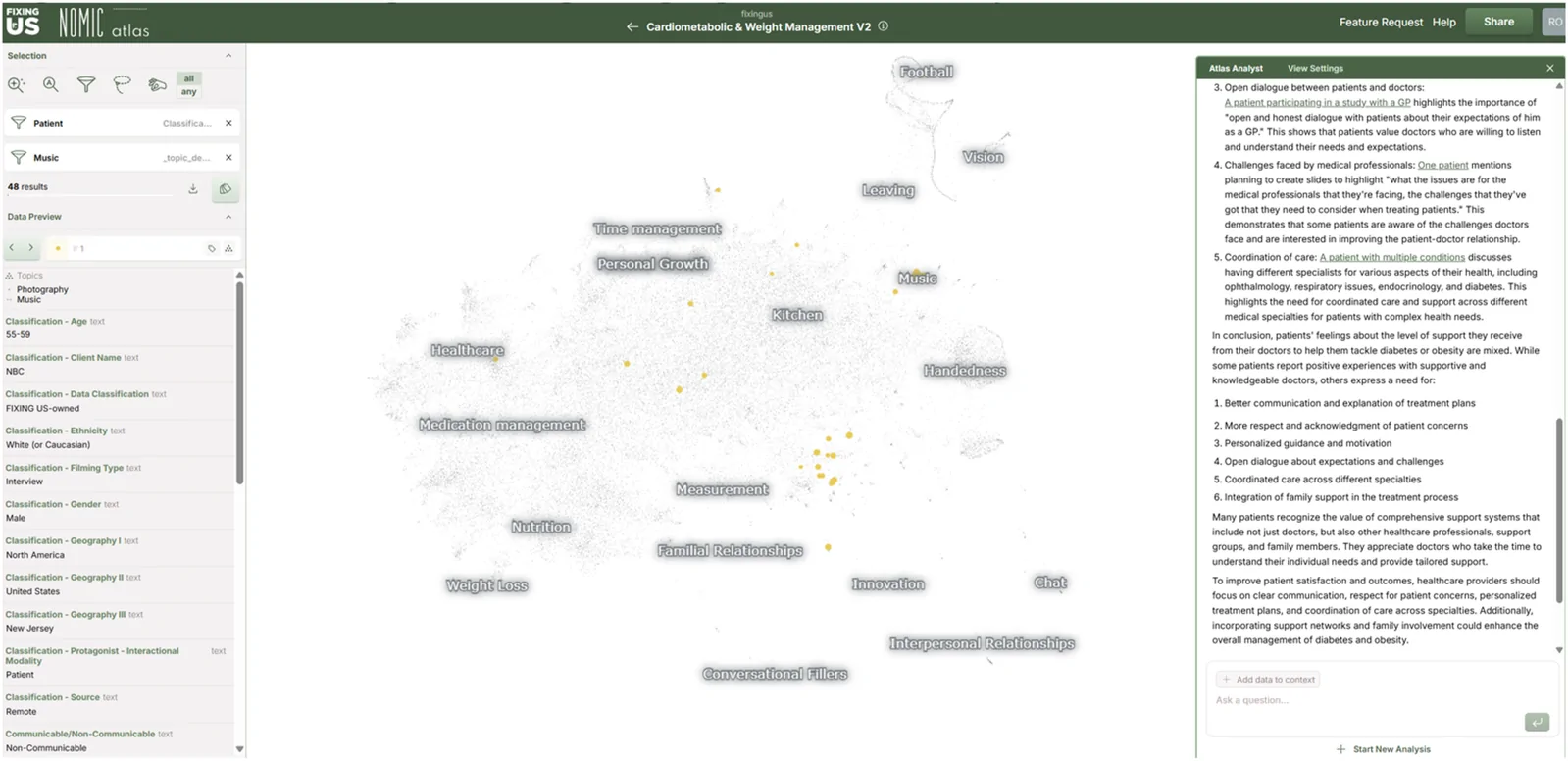
Authentic Intelligence's large language model user interface. FIXING US/NomicAI Atlas. The user interface for Authentic Intelligence enables users to ask questions to an extensive repository of transcribed filming, interviews and other forms of captured footage featuring patients, family members, caregivers, physicians, HCPs and other stakeholders so they can elicit qualitative AI-generative responses. Its basic-looking interface is geared towards enabling users to ask questions and to identify what parts of the datasets support the output but earlier versions of tools like this do not focus on governance considerations that will be discussed later in the paper.

Using generative-AI LLMs to elicit insights from authentic content to power wider diffusion and embedding of patient voices is another new frontier of health innovation (135). By creating datasets from raw footage, transcribed and framed within trained tags and classifications, it is possible to elicit more forms of qualitative information for new diffusions across the innovation system and mass personalization at previously impossible scale but it is important to take steps to minimise bias (whether due to how questions in captured footage are designed, interpreted and cognitive biases during capture) (136). Tools like *Authentic Intelligence* seek to address this by using transcribed raw footage captured using techniques designed to reduce bias and increase contextual and nuanced information. The composition of the source footage is illustrated in Figure 6.

**Figure 6:**
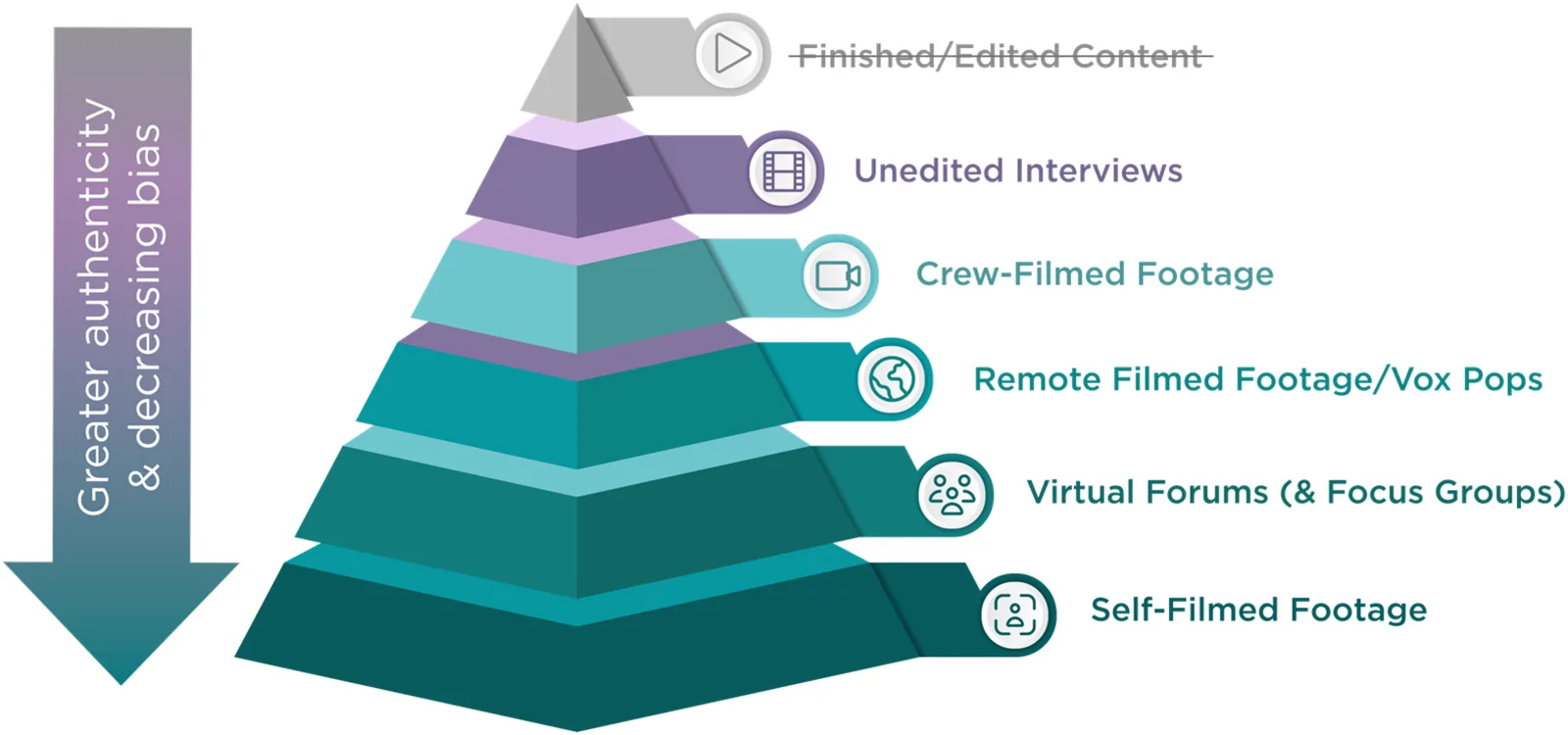
Composition of filmed footage collected for authentic intelligence (excluding curated/edited footage). FIXING US/fixingus.com. The final edited and deployed authentic content is a modest proportion of the total footage collected using the different methods and techniques for capturing filming, and it is only the raw footage (excluding the final authentic content) that would be transcribed and utilised for LLMs in order to ensure authenticity, increased contextual and nuanced information and minimised bias and misrepresentation.

New AI-powered technologies enable huge numbers of narratives to be analysed to identify patterns at scale with mass personalisation (137) that can be used in any stage of the HIS Framework. This attempted shift from “curated” to more “authentic” datasets can help organisations integrate authentic content rapidly, continually, efficiently and at scale—potentially accelerating value-creation, compressing timelines, improving outcomes, and potentially extending and bringing forward value-capture.

However, human oversight and empathy is crucial to ensure AI augments, and does not replace, the process of listening to patients. The value of patients' voices lies in its authenticity and emotional impact; if AI is used to generate synthetic “patient-like” stories without real experience, it would undermine trust. Frameworks for “ethical AI in patient-engagement” are being established (138) which emphasise using AI to amplify real voices, ensure representativeness, and flag insights so humans interpret and apply them appropriately.

Several governance considerations warrant emphasis before AI-enabled diffusion of authentic content could be recommended at scale and tools like *Authentic Intelligence* will need advancements that address these considerations. First, representativeness and bias: datasets built from filmed patient experiences will over-represent patients who are willing and able to be filmed, so systematic auditing of demographic and clinical representativeness is needed to avoid amplifying some voices while silencing others (136). Second, explainability: outputs derived from LLMs should be traceable to the underlying source narratives, so that users can verify that generated insights faithfully reflect what patients actually said and so that synthetic or hallucinated “patient-like” content can be detected and excluded (132). Third, consent and data governance: patients whose narratives are transcribed into datasets should provide informed consent covering secondary AI-enabled uses, with clear boundaries on downstream applications (138). Fourth, validation: claims that AI-derived insights improve decision quality, timelines or outcomes remain largely untested and should be evaluated prospectively using the operationalisation pathways proposed in this paper. Until such evidence accumulates, AI-enabled applications should be regarded as useful and promising but relatively unvalidated extensions of the framework.

The synergy of authentic content and AI is an emerging area that may amplify diffusions of large numbers of patients’ voices. AI can operationalise the “soft power” of storytelling, converting it into a resource that can be queried, quantified, and rapidly mobilised to inform decisions across all stages of the HIS Framework, with potential to find new ways to embed patients' voices, further accelerate timelines and increase the ROI so patients can access innovations earlier and new efficiencies. As long as ethical safeguards and human empathy guide its use, AI could significantly augment how patient voices are embedded in innovation systems.

### 4.3 Operationalising the HIS framework for empirical validation

If the HIS Framework is to progress from conceptual model to tested theory, its core constructs require explicit operationalisation. “Authentic content” can be operationalised through fidelity criteria derivable from the definition advanced in this paper (real patients or stakeholders; no actors, scripts, staging or re-enactment; editing that preserves the integrity of captured footage), together with exposure variables of dose (the volume and duration of content deployed), reach (the number and categories of stakeholders exposed) and, critically, timing (the innovation stage at which diffusion begins). “Stakeholder-engagement” can be measured through the number and diversity of stakeholder categories actively participating, the frequency and depth of interactions, the emergence of co-creation behaviours (participants becoming recruiters, sponsors or distributors), and established implementation outcomes such as acceptability, adoption, penetration and sustainability (56). “Value-creation” should be attributed into stage-specific endpoints rather than treated as a single construct.

Proposed stage-specific endpoints include: for clinical trials, time-to-full-enrolment, retention and completion rates, the number of substantial protocol amendments and cycle time (71, 74); for regulatory and HTA stages, the volume of clarification queries and the interval between marketing authorisation and reimbursement guidance; for deployment, time-to-formulary inclusion and the number of markets activated per unit of time; for HCP engagement and patient access, prescribing initiation, adherence and persistence measures (for example, proportion of days covered) and patient-experience scores; and, economically, expected net present value models of the kind demonstrated by Levitan and colleagues (73).

The HIS Framework also implies testable mediators and moderators. Candidate mediators (the mechanisms through which authentic content is proposed to exert its effects) include perceived authenticity, narrative transportation and identification (15), empathy, trust and self-efficacy (75). Candidate moderators include the timing of deployment (the central “leftward shift” proposition), therapeutic area and disease burden, jurisdictional regulatory constraints, stakeholder type and baseline brand or disease awareness. The leftward-shift proposition potentially yields a falsifiable prediction: for comparable assets, programmes deploying authentic content at or before the start of clinical development should show larger cumulative gains in engagement and value endpoints than programmes deploying it later, with effect sizes diminishing as deployment moves later in the lifecycle.

Appropriate designs for such tests include stepped-wedge or cluster designs across trial sites or country affiliates, interrupted time-series and difference-in-differences comparisons against matched programmes, and prospective registries of authentic content deployments recording dose, timing, reach and outcomes, complemented by qualitative process evaluation to examine mechanisms. Such studies would allow the basic and enhanced trajectories of the HIS Framework to be estimated empirically rather than illustrated conceptually. This contrast is illustrated in Figure 2.

### 4.4 Identifying limitations and the need for future research

Notwithstanding the consistency of the findings reviewed, this study has limitations that should be acknowledged. First, much of the evidence is compiled from case studies and reports rather than controlled primary research and this raises the possibility of confounding factors influencing outcomes. Innovators deploying authentic content may be simultaneously implementing increased funding or organisational enhancements that contribute to positive results. The attribution paradox remains, even when improved outcomes from authentic content are evident, and isolating a singular effect is challenging in complex systems. Future research should strive for rigorous methods to quantify the incremental impact of patient voice interventions on innovation outcomes.

Secondly, publication bias or selective reporting is likely when supporting the hypothesis. Case studies of successful patient-centric initiatives are likely to get published whilst efforts that did not yield benefits are less likely to be reported. Literature on related topics is still emerging and there is a need for studies that quantify specific impacts, outcomes and ROI metrics at different times so more can be learned about latency. Longitudinal research is necessary to see if embedding patient voices leads to sustained changes and to analyse both successful and unsuccessful case studies.

Thirdly, some of the illustrative examples presented (including the FIXING US broadcast series, Authentic Intelligence platform and any campaign initiatives in which the author's organisation participated) are connected to the author, and the HIS Framework itself is the author's creation. Although the corpus of evidence and case studies have no connection with the author, aspects in proximity with the author provide practical insight but introduces a risk of interpretive bias and unintentional self-validation. To mitigate this, these examples have been framed as practice-based observations rather than validated findings and are distinguished from independently conducted, peer-reviewed research such as the CTTI financial modelling (73), the evaluation of patient input into NICE appraisals (87) and published trial-level studies (74, 124). Independent replication of the framework's propositions by researchers with no stake in its adoption is an essential next step, and the operationalisation pathway set out above is intended to enable precisely such third-party evaluation.

It is also important to note that while there is evidence across different stages, the breadth of therapy areas covered is uneven and, whilst certain fields feature heavily in literature, other fields might have different dynamics and lack visibility. Future work should examine if authentic content has similar impacts in diverse health contexts and how authentic content impacts different therapies.

As AI and LLM advancements continue and more solutions like *Authentic Intelligence* emerge, it will be useful to assess outcomes from AI-powered diffusions from authentic content in terms of both favourable outcomes and potential pitfalls to help shape processes, best practices, policy, ethics and calibration of the most effective tools.

Finally, this arena is also likely to benefit from mechanistic studies that help us understand of *how* exactly authentic content exerts effects, how the scope of the stakeholder-mechanism can be affected and how psychological and communication science approaches can influence different facets of authentic content in addition to tracking temporal changes in influence of authentic content over time, individually and at scale.

On the other hand, the evidence assembled in this paper provides a reasonable basis for organisations to pilot the integration of authentic content across stages of health innovation while empirical validation proceeds. Despite the limitations, and even though precise ROI measures need refinement, the direction of the associations observed is consistently positive. Organisations need not wait for perfect metrics to trial a promising innovation mechanism, provided such initiatives are accompanied by the kinds of measurement approaches proposed above. As the industry evolves, patient voices and authentic content may increasingly be viewed as a complementary form of real-world evidence.

There is an opportunity to normalise and elevate the status of the patient voice in innovation and authentic content is a powerful form and mechanism for this. There is more work ahead to fully understand and maximise the value of authentic content in health innovation but the findings of this paper support the case for a move from “anecdote” to “evidence” (137) and highlight an opportunity for enhanced engagement, stronger collaboration, better economic returns and improved patient outcomes in terms of faster and more efficient access.

## 5 Conclusion

This paper has hypothesised that authentic content operationalises the patient voice as a catalyst for improved engagement, accelerated innovation, and greater value-generation. The evidence assembled—largely observational, case-based and industry-derived, but consistent in direction—supports the plausibility of the HIS Framework and its proposition that diffusions of authentic content are associated with accelerated progression of development, increased engagement, greater economic value and improved patient outcomes.

There is a strong conceptual case for authentic content to be deployed in earlier stages of health innovation due to the hypothesised knock-on effects from early impacts in the innovation process and how it shapes trajectory, receptivity and value-capture of health innovations, and how it can accelerate progression towards patient access and amplify legitimacy, effectiveness, ROI and system impact—to the extent that there is a case for redesigning the HIS Framework, shifting initial expansion of stakeholder-engagement and value-generation leftwards to the start of the Clinical Trials stage.

The findings suggest potentially high returns when patient voices inform clinical trials, regulatory submissions, and launch strategies, with reported improvements in success rates, timelines, and ROI. On this evidence, authentic content appears to be more than a “soft” contributor: it may function as a tangible economic driver of financial returns and patient outcomes, although this proposition requires empirical confirmation.

Authentic content also functions as a mechanism for deep stakeholder-collaboration and co-creation, helping to de-risk projects by aligning them with real-world needs and creating value that can go far beyond the value of any content created, and the addition of new AI-powered techniques to harness raw footage from authentic content further amplifies the ways that patient voices can be embedded across all stages at scale. Together, these aspects suggest that incorporating authentic content is not just a matter of corporate social responsibility or patient relations or communications but potentially a source of competitive advantage and an effective innovation driver.

This may be an evolving area and there may be need for further research and work to improve understanding but this study consolidates a consistent—though largely observational—body of supportive evidence, and the potential implications for healthcare organisations and health systems are considerable. Leaders should recognise that integrating authentic content is not merely a sentimental add-on or a communications consideration; the evidence reviewed suggests it may carry real functional and economic significance in modern health innovation.

If the hypothesis advanced here is supported by future empirical research, authentic content that embeds the patient voice may represent a high-value investment in the innovation process. It can strengthen the foundation of healthcare advancement by ensuring innovators remain patient-centric and innovations are grounded in patients’ needs and value. Moreover, earlier deployment is proposed to accelerate progression of development, with cumulative and multiplicative benefits that may result in considerably longer periods of effective commercial market exclusivity, more patients being able to access medicines faster, and providers being able to offer more efficient access as a result of greater scalability. In systems constrained by rising costs and eroding trust, the ability to embed the patient voice and translate the soft power of storytelling via authentic content into measurable gains in health innovation, financial returns and provision of care represents a significant strategic opportunity.

## Data Availability

The original contributions presented in the study are included in the article/Supplementary Material, further inquiries can be directed to the corresponding author/s.
